# Fast pulsatile blood flow measurement in deep tissue through a multimode detection fiber

**DOI:** 10.1117/1.JBO.25.5.055003

**Published:** 2020-05-13

**Authors:** Renzhe Bi, Yao Du, Gurpreet Singh, Chris Jun-Hui Ho, Shuyan Zhang, Amalina Binte Ebrahim Attia, Xiuting Li, Malini Olivo

**Affiliations:** Singapore Bioimaging Consortium, Singapore

**Keywords:** diffuse speckle contrast analysis, diffuse correlation spectroscopy, laser speckle, deep tissue blood flow

## Abstract

**Significance:** Noninvasive *in vivo* fast pulsatile blood flow measurement in deep tissue is important because the blood flow waveform is correlated with physiological parameters, such as blood pressure and elasticity of blood vessels. Compromised blood flow may cause diseases, such as stroke, foot ulcer, and myocardial ischemia. There is great clinical demand for a portable and cost-effective device for noninvasive pulsatile blood flow measurement.

**Aim:** A diffuse-optics-based method, diffuse speckle pulsatile flowmetry (DSPF), was developed for fast measurement (∼300  Hz) of deep tissue blood flow noninvasively. To validate its performance, both a phantom experiment and *in vivo* demonstration were conducted.

**Approach:** Over the past two decades, single-mode fibers have been used as detection fibers in most diffuse-optics-based deep tissue blood flow measurement modalities. We used a multimode (MM) detection fiber with a core size of 200  μm for diffused speckle pattern detection. A background intensity correction algorithm was implemented for speckle contrast calculation. The MM detection fiber helped to achieve a level of deep tissue blood flow measurement similar to that of conventional modalities, such as diffuse correlation spectroscopy and diffuse speckle contrast analysis, but it increases the measurement rate of blood flow to 300 Hz.

**Results:** The design and implementation of the DSPF system were introduced. The theory of the background intensity correction for the diffused speckle pattern detected by the MM fiber was explained. A flow phantom was built for validation of the performance of the DSPF system. An *in vivo* cuff-induced occlusion experiment was performed to demonstrate the capability of the proposed DSPF system.

**Conclusions:** An MM detection fiber can help to achieve fast (∼300  Hz) pulsatile blood flow measurement in the proposed DSPF method. The cost-effective device and the fiber-based flexible probe increase the usability of the DSPF system significantly.

## 1 Introduction

Blood flow is an important indicator of human health status. Compromised blood flow may cause diseases, such as stroke,^1^ foot ulcer,^2^ and myocardial ischemia.^3^ Several optical technologies have been developed for blood flow measurement. Laser speckle contrast imaging (LSCI) methods^4^^,^^5^ have been developed for wide-field blood flow imaging of superficial tissue (∼1  mm).^5^ It is a powerful tool for visualization of blood vessels. However, the penetration depth limits its applications in deep tissue blood flow measurement. Since biological tissue is a highly scattering medium for light, several diffuse optical methods have been developed for blood flow measurement in deep tissue (up to ∼15  mm).^6^ Diffuse correlation spectroscopy (DCS)^6^^–^^9^ is one of the first noninvasive optical methods; it has been used to monitor deep tissue blood flow in human muscle,^10^ brain,^11^ and breast cancer.^12^ DCS has many advantages including noninvasiveness, portability, and high temporal resolution (up to ∼100  Hz).^6^ However, there are challenges for DCS systems. To acquire the intensity temporal autocorrelation function $G_{2}(\tau )$, a single-mode (SM) fiber and a high-sensitivity single-photon counting avalanche photodiode (SPC-APD) have to be used for photon detection. To extend DCS into multiple channels, each detection channel requires one SPC-APD, which increases the hardware cost significantly. Diffuse speckle contrast analysis (DSCA) was developed as a cost-effective method for deep tissue blood flow measurement.^13^^–^^16^ DSCA uses the relationship between the speckle contrast of the diffused laser light and the blood flow index (BFI) in DCS to achieve a level of deep tissue blood flow measurements similar to that of DCS.^13^^,^^17^^,^^18^ The fiber-based DSCA system can be extended into multiple channels without significant additional cost.^17^ Several other deep tissue blood flow measurement modalities based on diffused laser speckle detection have been developed. Speckle contrast optical tomography^19^^,^^20^ achieves three-dimensional imaging of deep tissue blood flow. Diffuse speckle contrast flowmeter^21^ achieves a wearable sensor for cerebral blood flow monitoring in small animals.

Most of the abovementioned optical technologies work at a blood flow measurement rate within 10 Hz. Recently, fast pulsatile blood flow measurement in deep tissue has gained more popularity.^22^^,^^23^ The waveform of blood flow within each cardiac cycle is similar to the photoplethysmogram (PPG) waveform, but it has significantly different features.^22^ Many physiological parameters, such as blood pressure and blood vessel elasticity, are correlated with the two waveforms.^22^

To measure pulsatile blood flow in deep tissue, a DCS system based on a real-time software correlator that achieved a BFI measurement rate of 100 Hz was reported.^23^ Another method, speckle plethysmography, was developed for fast measurement of *in vivo* blood flow waveform and PPG waveform.^22^^,^^24^^,^^25^

The SM detection fiber is the main reason that fiber-based DSCA system cannot achieve fast blood flow measurement.^13^^,^^17^^,^^26^ A CCD camera is utilized as the detector in the fiber-based DSCA system. In each frame, only one speckle can be captured from one SM fiber. DSCA needs ∼60 speckles to calculate the diffused speckle contrast temporally. Therefore, the measurement rate of fiber-based DSCA is one sixtieth (1/60) of the CCD frame rate.

We introduced a method for fast pulsatile blood flow measurement in deep tissue based on a multimode (MM) detection fiber. Compared with free-space methods, fiber-based diffuse optical methods are more popular in biomedical applications because of their flexibility and robustness.^27^^–^^29^ Some researchers in DCS-related fields might hold the opinion that MM detection fiber will reduce the temporal coherence length of the laser light. In fact, a static MM fiber does not affect the temporal coherence of the laser. Many DCS systems use MM fibers with a core size of 200  μm as source fibers, which do not reduce the temporal coherence length of the delivered laser light.^10^^,^^30^ Although MM fibers do not decay $G_{2}(\tau )$ faster, they decrease the sensitivity to $G_{2}(\tau )$ when they collect laser light into a single element sensor, such as APD.^31^ DCS extracts BFI from $G_{2}(\tau )$ and thus uses SM detection fibers in most cases. However, DSCA does not need to acquire $G_{2}(\tau )$, but it uses the contrast information from the diffused laser speckles to measure the deep tissue blood flow. Therefore, it is possible to make use of an MM detection fiber with a multipixel photon detector, such as a CCD camera, to measure deep tissue blood flow. An MM detection fiber delivers multiple speckles into the CCD sensor, which allows the diffuse laser speckle contrast to be calculated spatially at each frame. Consequently, it makes the measurement rate of deep tissue blood flow the same as the CCD frame rate.

To distinguish the proposed method from the previously reported fiber-based DSCA system, it will be referred to as diffuse speckle pulsatile flowmetry (DSPF). The proposed DSPF system achieves 300 Hz simultaneous measurement of deep tissue blood flow and PPG waveforms. Both phantom validation and *in vivo* blood flow measurement are demonstrated. The portable and flexible fiber-based probe can be easily used on any surface of the human body. The instrumentation is simple and cost-effective. According to our knowledge, DSPF is one of the fastest noninvasive methods for deep tissue blood flow measurement.

## 2 Theory

It has been discussed that most optical modalities for blood flow measurements, including DCS, DSCA, LSCI, and laser Doppler flowmetry, examine different aspects of the decaying temporal autocorrelation function of the scattered light.^32^ In DCS, the BFI is usually obtained through model fitting of the electric field temporal autocorrelation function $G_{1}(\tau )$.^33^ In semi-infinite medium, $G_{1}(\tau )$ is given as^34^

$$G_{1}(r,\tau )=\frac{3\mu_{s}^{\prime }}{4\pi }[\frac{\exp(-k_{D}(\tau )r_{1})}{r_{1}}-\frac{\exp(-k_{D}(\tau )r_{2})}{r_{2}}],$$ (1)

where r is the source–detector (s–d) separation of the optical fiber probe, $k_{D}(\tau )=\sqrt{3\mu_{s}^{\prime }\mu_{a}+\alpha \mu_{s}^{\prime 2}k_{0}^{2}\langle r^{2}(\tau )\rangle }$, where $\mu_{s}^{\prime }$ is the reduced scattering coefficient, $\mu_{a}$ is the absorption coefficient, and α is the fraction of dynamic photon scattering events in the medium. $r_{1}=\sqrt{r^{2}+z_{0}^{2}}$, $r_{2}=\sqrt{r^{2}+{\left(z_{0}+2z_{b}\right)}^{2}}$, $z_{0}=1/\mu_{s}^{\prime }$, $z_{b}=2(1-R_{\mathrm{eff}})/3\mu_{s}^{\prime }(1+R_{\mathrm{eff}})$, where $R_{\mathrm{eff}}$ represents the effective reflection coefficient. $\left\langle r^{2}(\tau )\right\rangle$ is the mean square displacement of the moving particles after a delay time of τ.^17^^,^^34^ The Brownian motion model is the most popular model in DCS; it defines $\langle r^{2}(\tau )\rangle =6D_{b}\tau$, where $D_{b}$ is the effective diffuse coefficient.^34^ It is not practical to separate α and $D_{b}$ in most cases; thus $\alpha D_{b}$ is commonly taken as the BFI in DCS practice.

In DSCA, the speckles formed by the diffused laser light are used to assess the deep tissue blood flow. Laser speckle contrast, which is defined as K=σ/⟨I⟩, where σ is the standard deviation and ⟨I⟩ is the mean value of the pixels in the speckle pattern, is linked with $G_{1}(\tau )$ and $\alpha D_{b}$ by the following equation:^5^

$$K^{2}(T)=\frac{2\beta }{T}\int_{0}^{T}(1-\tau /T){\left[g_{1}(\tau )\right]}^{2}d\tau ,$$ (2)

where T is the exposure time of the CCD camera, β is the coherence factor determined by the ratio between the detector pixel size and the speckle size under the illumination of polarized light, and $g_{1}(\tau )$ is the normalized electric field autocorrelation function.

**Fig. 1:**
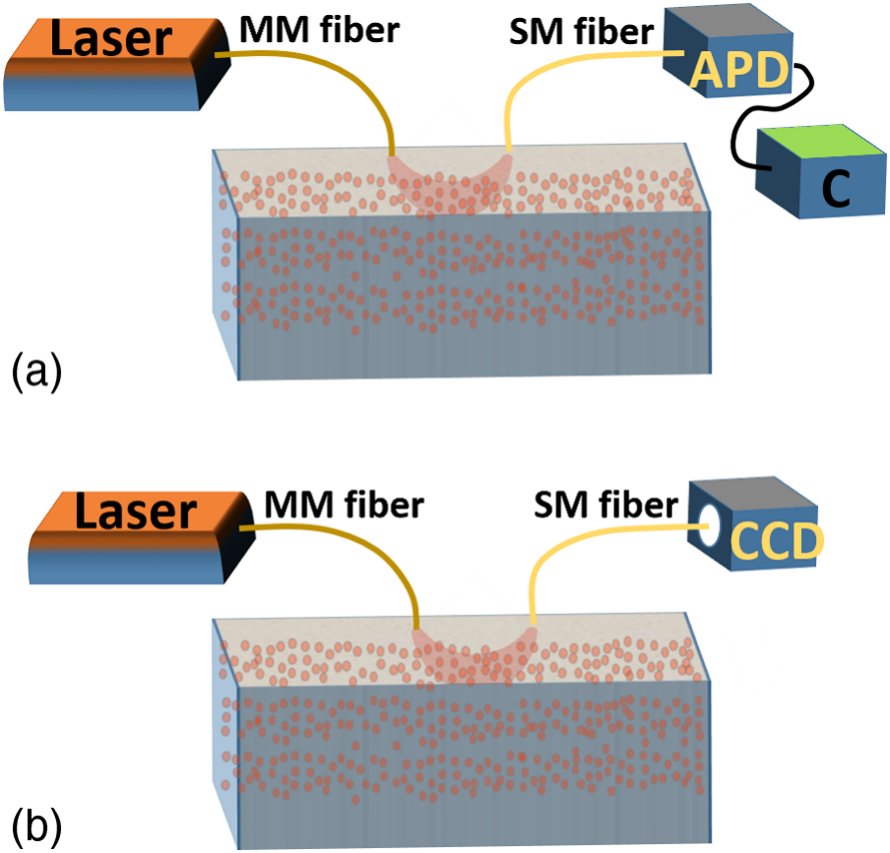
(a) Schematic of a DCS system. APD, avalanche photodiode; C, correlator for $G_{2}(\tau )$ calculation (it could be a hardware correlator or software correlator). (b) Schematic of a fiber-based DSCA system.

It has been shown that $1/K^{2}$ is linearly correlated to $\alpha D_{b}$ over the physiological range.^17^^,^^32^^,^^35^ Therefore, $1/K^{2}$ is used as BFI in DSCA for deep tissue blood flow measurement. A simple comparison between DCS and fiber-based DSCA systems is shown in Fig. 1.

The use of an SM detection fiber in DSCA systems limits the number of speckles acquired per frame. Only one single speckle can be captured at each CCD frame from one SM fiber.^17^ Typical DSCA systems need ∼60 frames to calculate $1/K^{2}$ temporally.^17^ Therefore, the measurement rate of BFI in DSCA is only 1/60 of the CCD frame rate.

There might be two main reasons that DSCA did not use MM fibers for detection: (1) DSCA is developed by following the convention of DCS, but DCS suggests not using MM detection fibers and (2) the speckle pattern output by an MM fiber is not an ideal speckle pattern and cannot be directly used for spatial speckle contrast calculation.

The intensity distribution of an ideal speckle pattern can be described as^36^

$$I(x,y)={\left|A(x,y)\right|}^{2}={\left|\frac{1}{\sqrt{N}}\overset{N}{\underset{k=1}{\sum }}|a_{k}|e^{i\phi_{k}}\right|}^{2},$$ (3)

where A(x,y) is the amplitude distribution and $|a_{k}|e^{i\phi_{k}}/\sqrt{N}$ is the k’th elementary phasor contribution. A fully developed speckle pattern has two statistical properties: (1) each contributing elementary phasor has independent amplitude and phase and (2) the phase follows a uniform distribution between [−π,π]. When N tends to be infinity, it could be derived that the probability density function of the intensity $p_{I}(I)$ is

$$p_{I}(I)=\frac{1}{\left\langle I\right\rangle }\cdot \exp(-\frac{I}{\left\langle I\right\rangle }),$$ (4)

where ⟨I⟩ is the mean intensity of the laser speckle pattern. The laser speckle contrast analysis is valid only when the intensity of all speckles in the speckle pattern follows the distribution described in Eq. (4). However, the intensity distribution of the laser speckle pattern output from the MM fiber tip is dependent on the beam shape. When a noncoherent light source is used, the intensity profile at the MM fiber tip is experimentally measured, which is similar to a Gaussian distribution.^37^ Therefore, the output speckle pattern can be expressed as

$$I_{\mathrm{MM}}(x,y)=I(x,y)\cdot I_{0}(x,y),$$ (5)

where $I_{\mathrm{MM}}(x,y)$ is the actual measured intensity map of the speckle pattern at the MM fiber tip, I(x,y) is the ideal speckle pattern, and $I_{0}(x,y)$ is the normalized background intensity profile of the MM fiber tip projected onto the CCD sensor when a noncoherent light source is used. Although $I_{\mathrm{MM}}(x,y)$ cannot be used for speckle contrast calculation, the ideal speckle pattern I(x,y) can be recovered by obtaining $I_{0}(x,y)$. Instead of using a noncoherent light source for illumination, $I_{0}(x,y)$ can be obtained by averaging a number of laser speckle patterns as well.

**Fig. 2:**
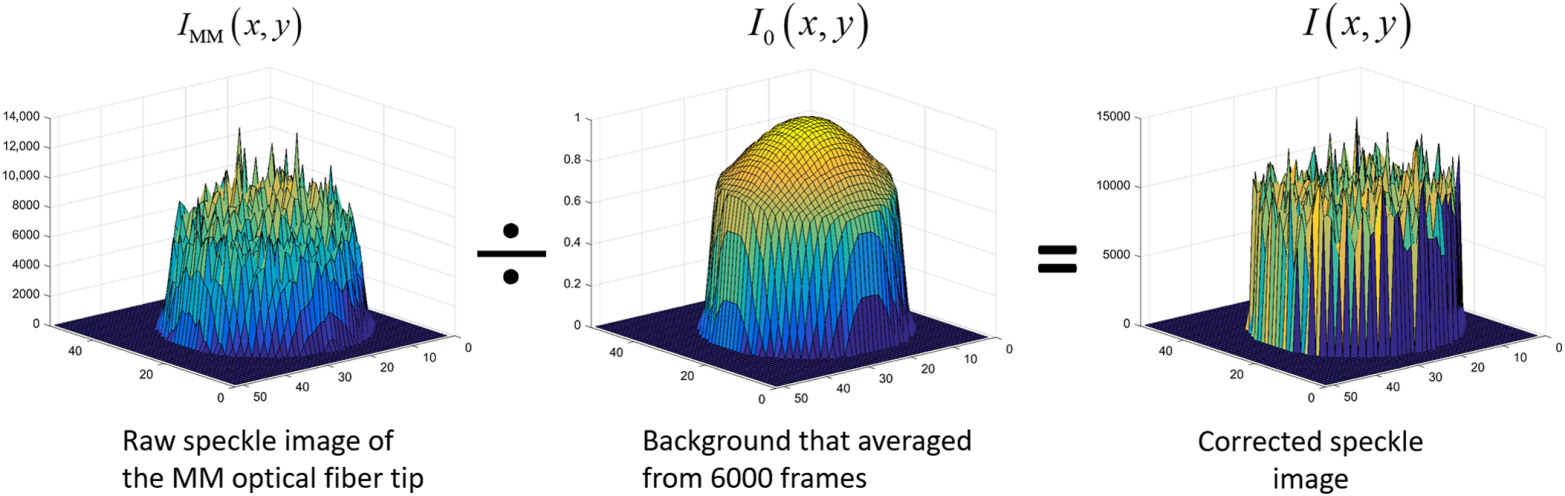
Illustration of the speckle pattern correction process. Around 6000 frames of speckle images (acquisition rate of 300 fps) are averaged to generate the background intensity profile. Then, the following raw CCD images will be divided by this background to obtain the corrected speckle pattern.

We average 6000 laser speckle images (at 300 fps imaging rate) and then normalize it to the maximum intensity. This measured background intensity profile will be used to correct every frame of raw speckle pattern from the MM fiber tip. The speckle pattern correction process is illustrated in Fig. 2.

After the raw speckle pattern is corrected using the method shown in Fig. 2, $1/K^{2}$ can be calculated spatially from   I(x,y) and serves as the BFI in the DSPF system. For an MM fiber with a core size of 200  μm and a CCD sensor with a pixel size of 5.6  μm, the speckle pattern contains about 1000 pixels, which provides sufficient statistical power. Therefore, instead of using ∼60 frames of single speckle images and the temporal speckle contrast, DSPF can generate BFI spatially from each frame of the CCD images. Consequently, it boosts the measurement rate of BFI to be the same as the CCD frame rate, which is ∼60 times faster than fiber-based DSCA.

## 3 Phantom Result

**Fig. 3:**
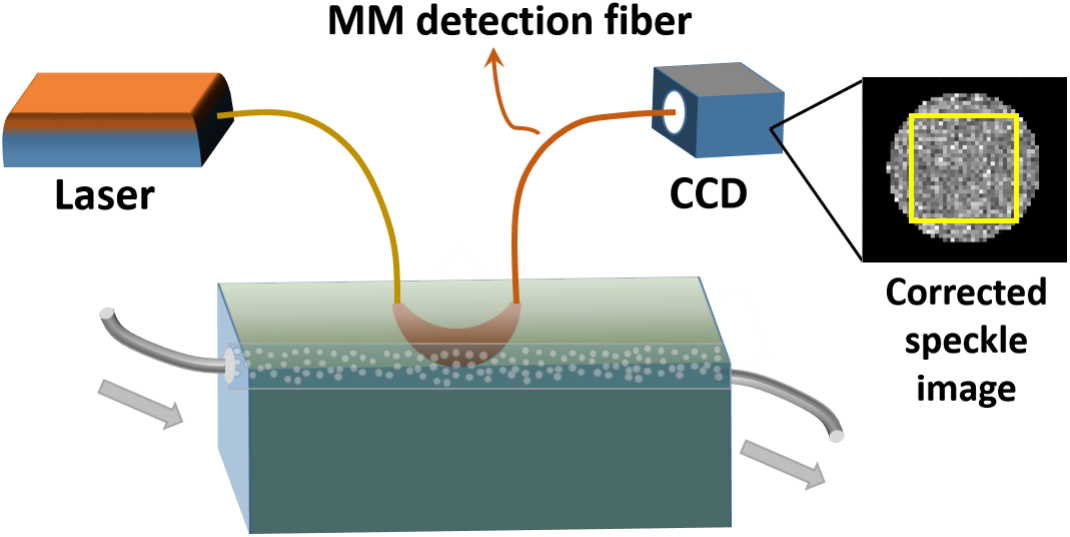
Schematic of the flow phantom experimental setup for DSPF system. Laser source is delivered by an MM fiber into the phantom. Another MM fiber with a core size of 200  μm is used for detection at a distance of 15 mm to the source fiber. The other side of the detection fiber is inserted into a camera, without the lens, touching the CCD sensor. An example of the corrected speckle image is displayed beside the CCD. The area used for $1/K^{2}$ calculation is marked by a yellow square.

To validate the performance of the DSPF system in deep tissue flow measurement, a flow phantom was built. A hollow tube filled with glass beads was embedded inside a solid scattering phantom body ($\mu_{a}=0.03\;{\mathrm{cm}}^{-1}$ and $\mu_{s}^{\prime }=8.6\;{\mathrm{cm}}^{-1}$). The tube was buried 6 mm underneath the phantom surface. Diluted Lipofundin (B. Braun, Germany) solution with a concentration of 0.6% was pumped through the tube by a peristaltic pump. The schematic of the experimental setup is shown in Fig. 3.

A long-coherence 785-nm laser (>5  m, DL785-100-S from CrystaLaser) was used as the light source and a compact industrial CCD camera (Flea3 GigE, Point Grey) was used as the detector. An MM fiber with a core size of 200  μm was used as the source fiber to deliver the laser light into the phantom. The other MM fiber with a core size of 200  μm functioned as the detection fiber. One end of the detection fiber was placed on the surface of the phantom, and the other end gently touched the CCD sensor. Considering the signal-to-noise ratio of the speckle image and the sensitivity to the flow, the typical exposure time of speckle detection in biomedical applications is 1 to 10 ms.^5^ An exposure time of 2 ms was used and the s–d separation was 15 mm for the phantom experiment. The CCD image acquisition rate was set at 300 fps. We experimentally estimated β [a parameter in Eq. (2)] of the MM detection fiber using a solid static phantom with the same optical properties as the flow phantom. The multiexposure approach we used was described by Parthasarathy et al.^38^ The experimental estimation of β was 0.19.

Before the flow measurement, 6000 images were acquired to generate the background intensity profile $I_{0}(x,y)$. Then every acquired frame was corrected against this background using the method illustrated in Fig. 2. An area of 25×25  pixels from the center of the corrected speckle pattern was used for $1/K^{2}$ calculation, as indicated by the yellow square box in Fig. 3.

**Fig. 4:**
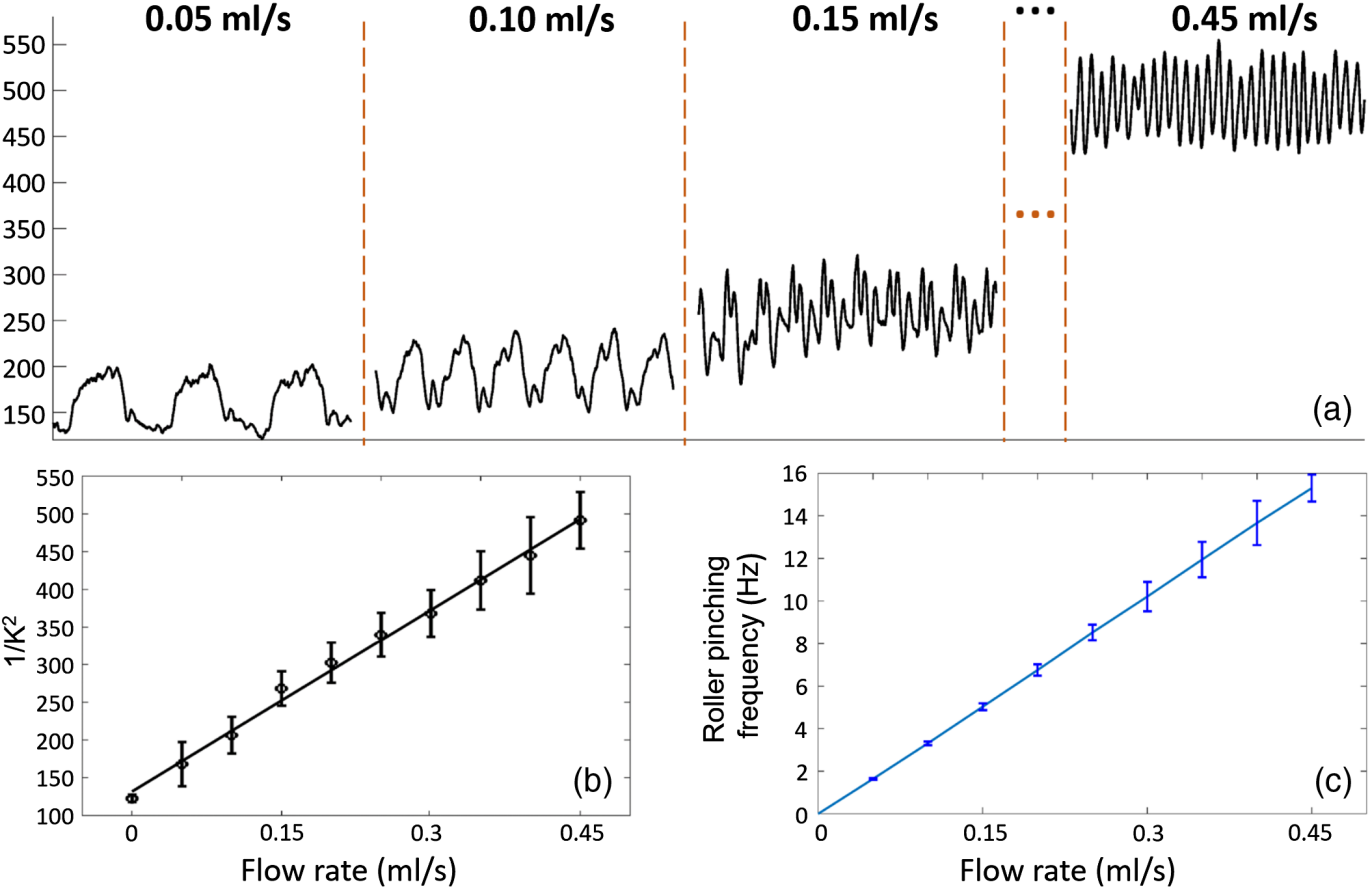
(a) Flow waveforms measured by DSPF system at different pumping rates. When the pumping rate becomes faster, the averaged flow measurement reading increases and the pinching frequency of the rollers also increases. (b) Averaged flow measurement at each flow rate. (c) Averaged pinching frequency at each flow rate. Pinching frequency is calculated from the peak-to-peak intervals of the raw flow waveform (Video 1, 3 MB, MP4 [URL: https://doi.org/10.1117/1.JBO.25.5.055003.1]).

During the experiment of flow measurement, the pumping rate was set from 0 to 0.45  ml/s at a step size of 0.05  ml/s. The peristaltic pump pumped liquid by pinching the rubber tube through the rotating rollers. A higher flow rate was achieved by increasing the pinching frequency of the rollers. Since the DSPF system provided a deep tissue flow measurement rate at 300 Hz, the flow waveforms at each pumping rate could be resolved clearly, as shown in Fig. 4(a).

At each flow rate, 10 s of flow measurement data (3000 data points) are averaged and plotted in Fig. 4(b), where a good linear relationship between the pumping rate and the averaged flow measurement readings can be observed (error bars are from the standard deviation of $1/K^{2}$). Since the DSPF system provides high temporal resolution, it resolves the periodical changes of the flow inside the phantom. Therefore, the roller pinching frequency can be calculated from the peak-to-peak time interval of the flow waveform. Figure 4(c) demonstrates a very good linear relationship between the flow rate and the measured pinching frequency (error bars are the standard deviation of pinching frequencies calculated from all peak-to-peak time intervals). Video 1 showcases the fast flow measurement using the phantom by a DSPF system.

## 4 *In Vivo* Result

In both DCS and fiber-based DSCA systems, the signal intensity fluctuation measured by the SM detection fiber is determined by $g_{1}(\tau )$, which cannot reflect the optical absorption without a long period of averaging. The proposed DSPF system uses an MM detection fiber with a core size of 200  μm, which captures hundreds of speckles at each frame. The averaged intensity of these speckles reflects the changes in the optical absorption. The measurement of the averaged intensity is very similar to the PPG, which is an indicator of the blood volume.^39^ By calculating the speckle contrast and mean intensity of the speckles from each frame, the DSPF system can simultaneously measure the relative changes of both blood flow and blood volume, respectively.

**Fig. 5:**
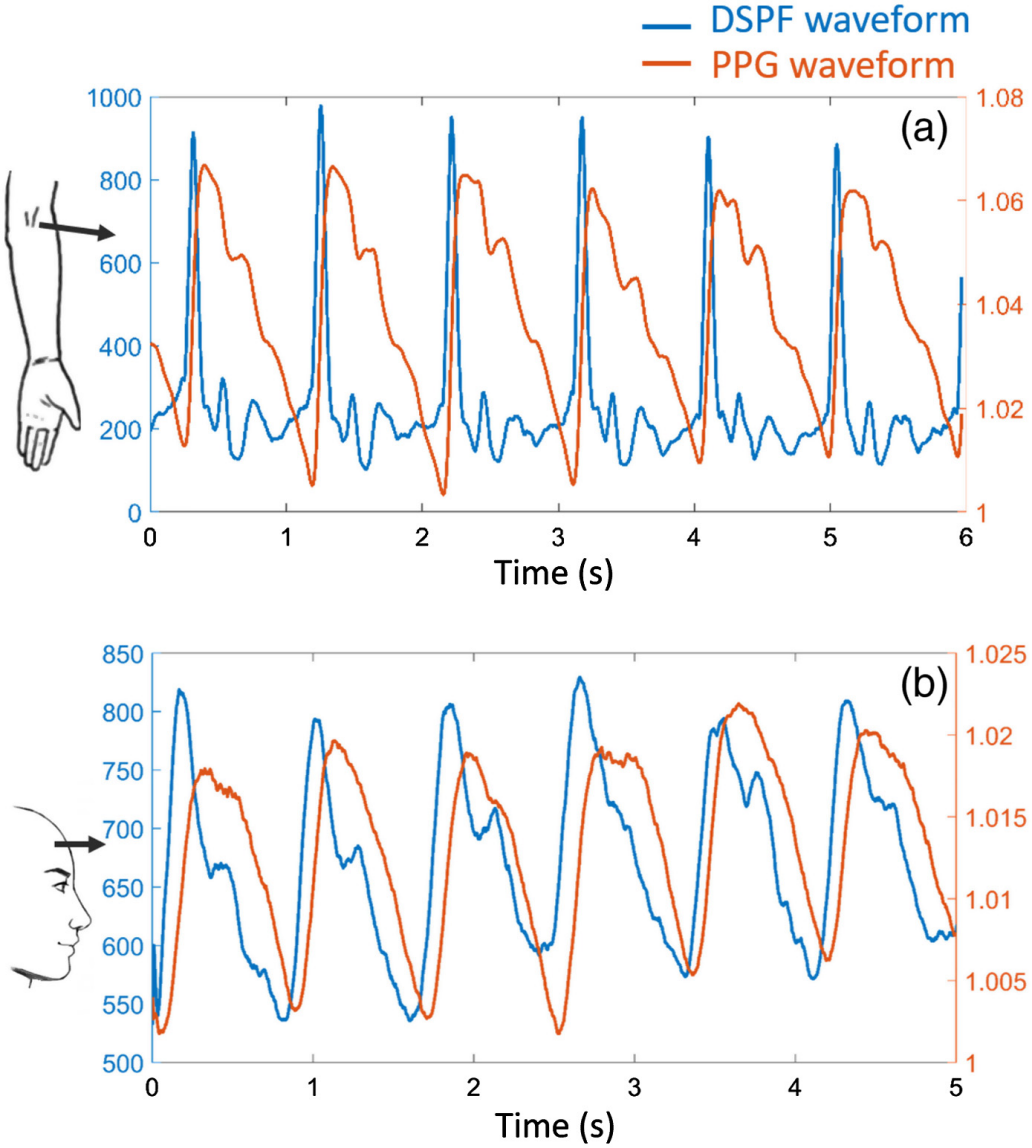
Simultaneous measurement of DSPF (blue) and PPG (orange) waveforms. Both (a) brachial artery (s–d separation: 15 mm and laser power: 4 mW) and (b) right prefrontal cortex (s–d separation: 25 mm and laser power: 20 mW) measurements are demonstrated. The exposure time of the CCD camera was 2 ms. The corresponding measurement locations are indicated on the left (Video 2, 980 KB, MP4 [URL: https://doi.org/10.1117/1.JBO.25.5.055003.2]).

The *in vivo* blood flow waveform (DSPF waveform), however, is different from the PPG waveform. Simultaneous measurements of both DSPF and PPG waveforms were performed on a healthy volunteer with informed consent. The brachial artery was measured with s–d separation of 15 mm and laser power of 4 mW; the right prefrontal cortex was measured with s–d separation of 25 mm and laser power of 20 mW. The CCD exposure time was 2 ms for both measurements. The results are shown in Fig. 5.

Unlike the PPG waveform, the DSPF waveform has sharper peaks and more features within each cardiac cycle. More interestingly, the peak of the blood flow is always in front of the corresponding peak of the blood volume. When the heart pumps blood into the aorta, the flow rate reaches its maximum first. The increased flow rate generates higher pressure on the artery wall. Consequently, the artery wall expands and contracts to transport the blood forward, resulting in local pulsatile volume change. We can observe this process from the simultaneous measurements of DSPF and PPG waveforms. A similar phenomenon has been reported using a nonfiber-based system.^22^ The flexible configuration of the fiber-based probe in the DSPF system enables measurement on any skin surface of the body. Video 2 showcases the real-time simultaneous measurements of the two waveforms on a healthy volunteer’s middle finger.

**Fig. 6:**
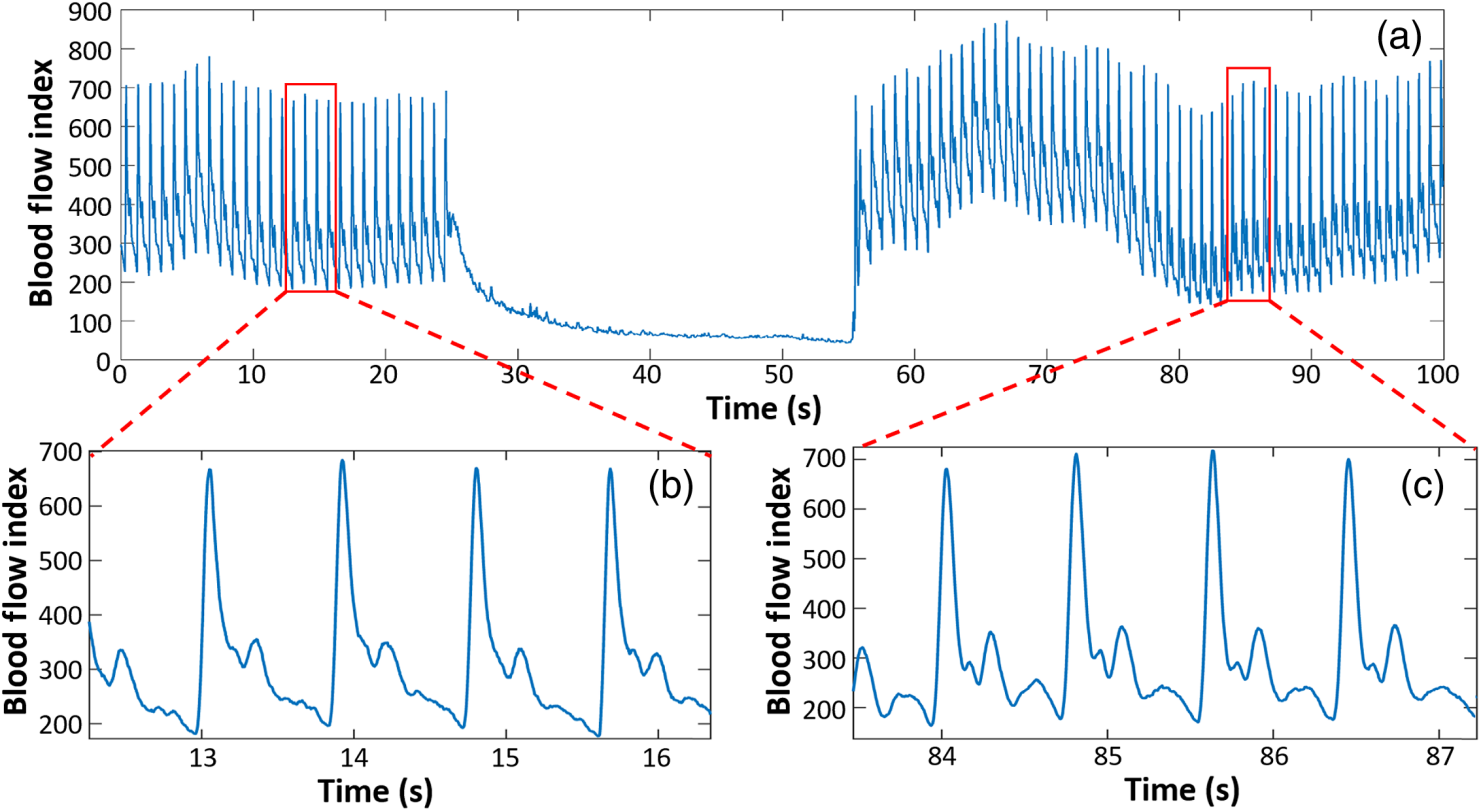
(a) Blood flow measurement of a healthy subject’s thumb during an arm-cuff-induced occlusion experiment. (b) Zoom-in view of the pulsatile blood flow waveform during baseline. (c) Zoom-in view of the pulsatile blood flow waveform during recovery.

To demonstrate DSPFs capability of measuring dynamic changes of *in vivo* blood flow, a cuff-induced occlusion protocol was designed. A healthy volunteer’s thumb was measured for 25 s in a rest state as a baseline. A pressure of 120 mm Hg was then applied to the arm cuff and held for 30 s. Finally, the cuff was deflated. The measurement continued for 45 s during the recovery period. The resultant DSPF waveform is shown in Fig. 6(a).

The pulsation of blood flow disappeared when the external pressure was exerted at 25 s. The overall BFI dropped dramatically during the occlusion period. A hyperemia reaction can be observed from Fig. 6(a) after the external pressure was removed at 55 s. The BFI dropped to the baseline level soon after 80 s. The DSPF waveforms at the initial baseline and the recovery phase are zoomed-in, as shown in Figs. 6(b) and 6(c).

## 5 Discussion and Conclusion

We present a DSPF system for fast (300 Hz) measurement of blood flow in deep tissue. The main difference between the DSPF system and existing methods, such as DCS and fiber-based DSCA systems, is the use of an MM fiber as a detection fiber. With an MM detection fiber and a CCD sensor, hundreds of speckles can be captured and corrected at each frame. This allows the diffuse speckle contrast to be calculated spatially from each CCD image. Therefore, the blood flow measurement rate is equal to the CCD frame rate. Limited by the SM detection fiber, a conventional fiber-based DSCA system acquires only one single speckle from each CCD image. It has to collect diffuse speckles through multiple frames (typically ∼60 frames) of images for contrast calculation, which leads to a much slower (∼1/60) blood flow measurement rate compared with the CCD frame rate.

The use of an MM detection fiber in the DSPF system allows for simultaneous measurement of the relative changes of blood flow and blood volume, as shown in the *in vivo* results. The simultaneous DSPF and PPG waveforms may contain important physiological information, such as blood pressure and blood vessel stiffness.

Another advantage of the DSPF system is its simple and cost-effective instrumentation. It shares the same hardware configuration as the tDSCA system,^17^ except it uses an MM detection fiber instead of an SM fiber. The implementation of the background intensity profile removal algorithm is straightforward. The flexible fiber probe can be easily adapted for any position on the skin in biomedical applications.

However, DSPF also has some disadvantages. Before each acquisition, the fiber probe needs to be fixed on the target, and it takes about 20 s to generate the output beam profile of the MM fiber. If the MM fiber shakes violently or moves to a different location, the background beam profile has to be recalibrated. The current design of the DSPF system includes one laser source at a single wavelength, which cannot provide oxygen saturation measurement from the PPG signal. In the future, a multiwavelength DSPF system will be designed.

In summary, the proposed DSPF system achieves fast noninvasive deep tissue pulsatile blood flow measurement at 300 Hz and the simultaneous relative measurement of both blood flow and blood volume. Its performance of deep tissue flow measurement is validated using a flow phantom. The *in vivo* measurement is also demonstrated. With its cost-effective hardware design and the flexible fiber-based probe, we envision the DSPF system will be tested in clinical studies in the near future.
